# The emerging role of histone lysine demethylases in prostate cancer

**DOI:** 10.1186/1476-4598-11-52

**Published:** 2012-08-06

**Authors:** Francesco Crea, Lei Sun, Antonello Mai, Yan Ting Chiang, William L Farrar, Romano Danesi, Cheryl D Helgason

**Affiliations:** 1 Experimental Therapeutics, British Columbia Cancer Research Centre, 675 West 10th Avenue, Vancouver, BC, Canada, V5Z 1L3; 2 Department of Internal Medicine, Division of Pharmacology, University of Pisa, Via Roma 55, 56100 Pisa, Italy; 3 Cancer Stem Cell Section, Laboratory of Cancer Prevention, Frederick National Laboratory for Cancer Research, 1050 Boyles Street, Building 560, Room 21-81, Frederick, MD 21702, USA; 4 Department of Drug Chemistry and Technologies, Sapienza University of Rome, P. le A. Moro, 5, 00185 Rome, Italy; 5 Department of Surgery, Faculty of Medicine, University of British Columbia, 910 West 10th Avenue, Vancouver, BC, Canada V5Z 4E3

**Keywords:** Prostate cancer, Epigenetics, Tumor-initiating cells, Histone demethylase, Androgen receptor

## Abstract

Early prostate cancer (PCa) is generally treatable and associated with good prognosis. After a variable time, PCa evolves into a highly metastatic and treatment-refractory disease: castration-resistant PCa (CRPC). Currently, few prognostic factors are available to predict the emergence of CRPC, and no curative option is available. Epigenetic gene regulation has been shown to trigger PCa metastasis and androgen-independence. Most epigenetic studies have focused on DNA and histone methyltransferases. While DNA methylation leads to gene silencing, histone methylation can trigger gene activation or inactivation, depending on the target amino acid residues and the extent of methylation (me1, me2, or me3). Interestingly, some histone modifiers are essential for PCa tumor-initiating cell (TIC) self-renewal. TICs are considered the seeds responsible for metastatic spreading and androgen-independence. Histone Lysine Demethylases (KDMs) are a novel class of epigenetic enzymes which can remove both repressive and activating histone marks. KDMs are currently grouped into 7 major classes, each one targeting a specific methylation site. Since their discovery, KDM expression has been found to be deregulated in several neoplasms. In PCa, KDMs may act as either tumor suppressors or oncogenes, depending on their gene regulatory function. For example, KDM1A and KDM4C are essential for PCa androgen-dependent proliferation, while PHF8 is involved in PCa migration and invasion. Interestingly, the possibility of pharmacologically targeting KDMs has been demonstrated. In the present paper, we summarize the emerging role of KDMs in regulating the metastatic potential and androgen-dependence of PCa. In addition, we speculate on the possible interaction between KDMs and other epigenetic effectors relevant for PCa TICs. Finally, we explore the role of KDMs as novel prognostic factors and therapeutic targets. We believe that studies on histone demethylation may add a novel perspective in our efforts to prevent and cure advanced PCa.

## Introduction: prostate cancer epigenetics

It has been estimated that 240,890 men in the US developed prostate cancer (PCa) during 2011 [1]. In western countries, PCa is the most common male neoplasm, and the second leading cause of male cancer-related deaths. Unlike other common neoplasms (lung and colon cancer), PCa incidence has increased in the past few years. PCa is a double-stage disease, which usually starts as a treatable and poorly aggressive neoplasm. Early PCa can be treated by a combination of surgery, radiation and hormonal therapy [2]. The last option includes castration and pharmacological disruption of androgen-receptor (AR) signalling, which is a major proliferation stimulus for prostate cells. After a variable time, PCa may evolve into an aggressive neoplasm, usually referred to as castration-resistant PCa (CRPC) [3]. CRPC is associated with metastasis to lung, brain, liver and bones in 90% of the cases, and displays an 18-month median survival [4]. CRPC is resistant to conventional treatments, and chemotherapy itself can only delay its progression. Currently, few molecular targets are available to treat CRPC [3], and few prognostic factors are associated with PCa recurrence and progression [5]. Thus, the identification of novel prognostic factors and therapeutic targets is highly needed in this field.

**Figure 1:**
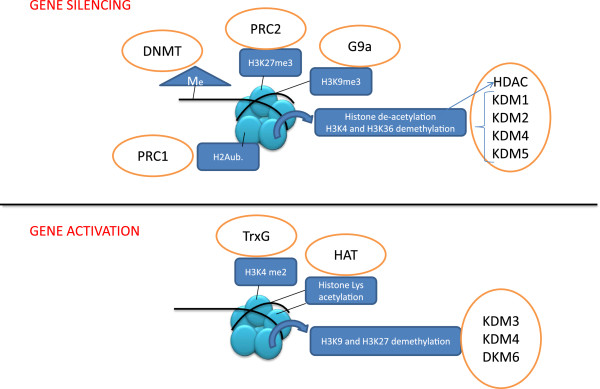
**Epigenetic mechanisms of gene silencing and activation.** Nucleosomes are composed of DNA (black strip) wrapped around 4 couples of core histones. Histone tails and DNA cytosines may be modified by several enzymes (orange circles), which add or remove epigenetic marks. Local gene function results from the combined interaction of all epigenetic enzymes. Me, methylation; K, lysine; ub, ubiquitination; DNMT, DNA methyltransferase; PRC, Polycomb repressive complex; G9a, histone H3K9 methyltransferase; HAT, histone acetyltransferase; HDAC, histone deacetylase; KDM, lysine-specific demethylases.

In the past decade, it has become increasingly evident that epigenetic gene regulation plays a crucial role in PCa initiation and progression. Epigenetics refers to all heritable changes which are not dependent on modifications of DNA primary sequence (summarized in Figure 1) [6]. The two classical epigenetic marks are DNA methylation and histone post-translational modifications (HPTMs). DNA methyltransferases (DNMTs) target cytosine residues, particularly on gene promoter regions, thereby triggering gene silencing. Several tumor suppressor genes are silenced by DNMTs in PCa, including pro-apoptotic, anti-metastatic and growth-inhibiting factors [7]. HPTMs orchestrate chromatin transcriptional activity, through a complex “histone code” [8]. The basic chromatin structure is represented by the nucleosome (Figure 1) consisting of 140-160 base pairs of DNA wrapped around a repetitive nucleosome core composed of four couples of histones H2A, H2B, H3 and H4. N-terminal histone tails protrude from this structure and may be targeted by several histone modifiers including methyltransferases/demethylases and acetyltransferases/deacetylases. Each modification may loosen or tighten local DNA-histone binding, thereby contributing to gene reactivation or silencing. For example, histone acetylation is always coupled to gene activation, while histone deacetylation leads to gene silencing [9]. Histone methylation may occur on different lysine residues, with opposite effects: histone H3-lysine 4 dimethylation (H3K4me2) and H3K36me2 are activating marks, while H3K27me2/3 and H3K9me2/3 are repressive marks [8].

HPTMs have been linked to PCa metastasis and/or androgen-independence by several studies. For example, specific histone deacetylases (HDACs) are over-expressed in androgen-independent PCa, and may contribute to chemo-resistance [10]. HDAC inhibitors are able to re-sensitize PCa cells to hormonal therapy [11], and are currently being tested on CRPC patients [12]. Polycomb (PcGs) and Trithorax (TrxGs) group genes encode for epigenetic modifiers with opposite roles in embryo development and cancer. Both PcG and TrxG proteins are organized into multimeric complexes, mediating specific HPTMs (Figure 1). In particular, Polycomb repressive Complexes (PRCs) are involved in gene silencing, through histone H2A ubiquitination (PRC1) and H3K27 trimethylation (PRC2) [9]. PcG members BMI1 and EZH2 contribute to PCa metastasis through their gene silencing function [13]. Mapping H3K27me3 on specific *loci*, a PRC2-dependent gene signature has been associated with poorer prognosis in advanced PCa [14]. PRC-dependent gene silencing may be counteracted by H3K4me2, mediated by TrxGs [9]. Interestingly, some members of this class are emerging as tumor suppressors in PCa [15].

In 2004, a human lysine-specific demethylase (KDM) was described for the first time [16]. Since then, several KDM isoforms were discovered, and characterized as epigenetic enzymes [17]. KDMs may counteract different histone methyltransferases (like PcG or TrxG members), thereby activating or repressing gene expression (Figure 1). Since HPTMs are known to orchestrate critical functions of PCa cells, it is conceivable that KDMs are key players of those processes. In this review, we will summarize emerging evidence on the role of KDMs in PCa progression, and will propose their use as prognostic factors and therapy targets.

## Histone demethylases and prostate cancer

Until the discovery of KDM1A (LSD1), histone lysine methylation had been long considered an irreversible epigenetic mark. KDM1A specifically demethylates H3K4me1/2 [16]. Currently, two KDM1 isoforms have been described (KDM1A and B); both are flavin-dependent demethylases with the same histone residue specificity [17]. Since H3K4me2 is an active mark, KDM1 favours gene silencing. However, when recruited by androgen receptor (AR), KDM1A loses its capability to demethylate H3K4me1/2 and instead catalyzes the demethylation of H3K9me1/2, two repressive marks, thus acting as a co-activator [18]. A second class of KDMs is represented by the Fe^2+^/oxoglutarate-dependent enzymes, containing a characteristic Jumonji C (JmjC) domain [19]. Those KDMs are divided into six clusters (KDM 2-7), most of which include at least two members [17]. Each cluster is characterized by one or more target histone residues. For example, KDM2 and KDM5 clusters are mainly involved in removing active marks (H3K4me2 and H3K36me2), thereby triggering gene silencing [20]. To the contrary, KDM3 and KDM6 mediate gene reactivation [21]. In particular, KDM3 genes remove H3K9me2, a repressive mark catalyzed by the putative oncogene G9a [22]. KDM6 removes Polycomb-dependent H3K27me3 [9], thereby playing a potential tumor-suppressive function in PCa. Finally, the KDM4 cluster includes both activating and repressive enzymes [17]. Due to the large number of family members exhibiting different functions, it is not surprising that KDMs may play oncogenic or tumor-suppressive roles in several neoplasms. For example, a census of frequently mutated cancer genes identified KDM5A, 5C and 6A are implicated in several malignancies, including acute myeloid leukemia, esophageal, renal and squamous cell carcinomas [18]. Since KDMs display a wide variety of “aliases” in the literature, we summarized in Table 1 the most common names for each gene discussed in this manuscript.

**Table 1:** **Formal names and aliases of KDMs cited in this manuscript. Aliases are from GeneCards website (**http://www.genecards.org)

**GENE NAME**	**ALIASES**
KDM1A	LSD1, BHC110, AOF2
KDM1B	LSD2, AOF1
KDM2A	JHDM1A, CXXC8, FBL7
KDM3A	TSGA, JHDM2A,JMJD1A2, JHMD2A
KDM3B	JHDM2B, NET22
KDM4A	JHDM3A, JMJD2
KDM4B	JHDM3B, JMJD2B
KDM4C	GASC1, JHDM3C, JMJD2C
KDM5A	JARID1A, RBBP2
KDM5B	JARID1B, RBBP2H1A, PLU1, CT31
KDM5C	JARID1C, SMCX, XE169
KDM6A	UTX
KDM6B	JMJD3
KDM7	JHDM1D
PHF8	JHDM1F, ZNF422

KDM1A, the most deeply studied lysine-specific demethylase, is a putative oncogene in several neoplasms including PCa [17]. Its oncogenic function partially resides on the ability to trigger Myc-dependent transcription [23], while inhibiting p53 pro-apoptotic function [24]. In PCa, KDM1A mainly acts as an AR co-activator [25]. In complex with AR, KDM1A, after histone H3 threonine 6 phosphorylation by protein kinase C β1 (PKCβ1) [26], changes its specificity from H3K4me1/2 to H3K9me1/2, thereby switching its role from co-repressor to co-activator [27]. Interestingly, pargyline blocked demethylation of H3K9me1/2 during androgen-induced transcription [25], and the tranylcypromine derivatives NCL-1 and NCL-2 [28] reduced androgen-dependent proliferation in PCa cells through KDM1A inhibition. In some circumstances, KDM1A may also act as an AR co-repressor. In the presence of high androgen concentrations, AR recruits LSD1 to mediate *AR* gene silencing [29]. This negative feedback loop is probably disrupted in CRPC, where low androgen levels favour AR over-expression. In addition, high KDM1A expression in primary PCa predicts higher risk of relapse after prostatectomy [30]. Thus, it is conceivable that KDM1A triggers androgen-dependent proliferation and recurrence after therapy. It is worth mentioning that some authors did not manage to confirm a significant correlation between KDM1A expression and PCa progression [31]. This may be due to smaller sample size, and/or differences in technologies employed. Other KDMs were identified as AR-co-activators (Table 2), but their role in PCa progression has not been clarified. One of them (KDM4C) co-operates with KDM1A to remove H3K9me marks, thereby activating AR targets [32]. Interestingly, KDM4C is required for cancer cell proliferation [33], and its expression is higher in CRPC, compared to hormone sensitive tumors and prostate hyperplasia [31]. Further studies are required to elucidate the relationship between AR, KDM1A/4C and PCa transition to an androgen-independent state, but these preliminary data indicate that those genes are promising therapy targets to inhibit early PC progression.

**Table 2:** **List of KDM genes described as relevant for PCa. For gene aliases, see** Table 1

**GENE**	**ROLE IN PROSTATE CANCER**	**REF.**
KDM1A	Oncogene, AR co-activator, associated with higher relapse risk	25-28
KDM2A	Putative tumor suppressor, reduced expression in PCa	41
KDM3A	Putative oncogene, overexpressed in PCa, AR co-activator	30, 33
KDM4A	Overexpressed in PCa	33
KDM4B	Overexpressed in PCa	33
KDM4C	Putative oncogene, overexpressed in CRPC, AR co-activator	30-32
KDM5B	Overexpressed in metastatic PCa, AR-co-activator	35
KDM5C	Putative oncogene, overexpressed in PCa, suppresses TGFB signalling	36
KDM6B	Putative oncogene, overexpressed in metastatic PCa	38
PHF8	Putative oncogene, overexpressed in PCa, mediates cell invasion	33

Some KDMs have been implicated in PCa metastatic spreading. A systematic knockdown of epigenetic enzymes in PCa cells identified PHF8 as a novel oncogene [34]. PHF8 contributes to gene activation through H3K9me1/2, H3K27me2, and H4K20me1 demethylation. It is over-expressed in PCa compared to normal prostate. PHF8 inhibition reduces proliferation of AR-positive and AR-negative PCa cells. In addition, PHF8 mediates PCa cell invasion and migration, two crucial steps of metastatic spreading. PHF8 silencing was associated with down-regulation of integrin-related genes. Integrin-dependent signalling is known to mediate PCa invasion [35]. Recent evidence indicates that members of the KDM5/JARID cluster may also be involved in PCa metastasis. KDM5B, which is significantly over-expressed in local and metastatic PCa, is an AR-co-activator [36]. KDM5C was shown to inhibit the transcriptional activity of SMAD3, a TGFβ-dependent transcription factor, independently of its enzymatic activity [37]. TGFβ signalling in PCa blocks early tumorigenesis, but paradoxically enhances metastatic spreading [38]. Thus, KDM5C may act as an oncogene in early PCa, but hinder metastatic spreading at later stages. Finally, the H3K27-specific demethylase KDM6B is progressively over-expressed in higher stage PCa [39]. H3K27 trimethylation is mediated by PRC2 component EZH2, which is up-regulated in metastatic PCa cells, and required for tissue invasion and blood dissemination [13,40]. Although it is not clear why metastatic PCa cells over-express two epigenetic proteins with opposite functions, one possibility is that a compensatory mechanism is activated in those cells. Alternatively, co-ordinated up-regulation of EZH2 and KDM6B allows for selective up- and down-regulation of specific target genes, depending on local adaptive needs of metastatic cells. Indeed, the metastatic process requires a certain degree of gene expression plasticity [41].

As shown in Table 2, other KDMs display up-regulation in PCa samples, compared to non-transformed cells [17,34]. KDM2A is down-regulated in PCa, where it may play a tumor-suppressive function through its role in maintaining genome integrity [28]. Very little is known on the specific cellular function of these genes in PCa. Future studies should dissect molecular pathways linked to KDM activation or inactivation, as well prospectively investigate KDM expression patterns in specific cancer subtypes (metastatic vs. primary; high vs. low grade; CRPC vs. hormone-sensitive PCa). These studies will allow us to identify the most promising therapeutic targets and prognostic factors.

## Histone demethylases as prognostic factors and therapy targets

**Figure 2:**
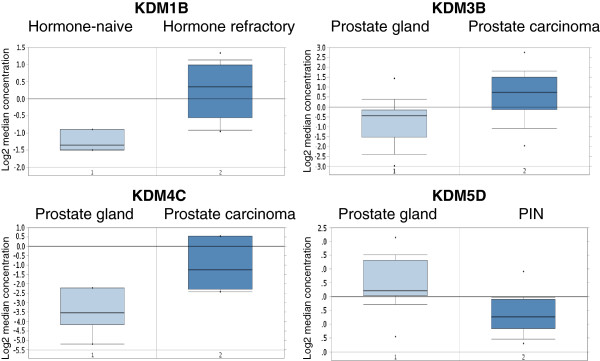
**KDM genes significantly modulated in PCa (Oncomine database).** Genes listed in Table 3 were queried in the Oncomine database. Significant associations are presented in this figure. PIN, prostatic intra-epithelial neoplasia. KDM1B, p < 0.001, fold-change, 2.746; KDM3B, p < 0.001, fold-change, 2.516; KDM4C, p < 0.001, fold-change, 5.89; KDM5D, p < 0.001, fold-change, -2.178. With the exception of KDM4C, we found one significant correlation per gene. For KDM4C, we report the most significant correlation, while the other one (tumor grade) is only quoted in the main text.

As summarized in the previous paragraph, emerging evidence indicates that KDMs play key roles in specific steps of PCa progression and particularly in transition from an androgen-dependent to an androgen-independent state, as well as in metastatic spreading. Apart from a few exceptions (KDM1A, PHF8) the molecular function of KDMs in PCa cells remains obscure. A useful cancer biomarker may predict cancer risk, tumor progression or response to a specific therapy [42]. Only in the first case, is over-expression in cancer vs. normal cells required to obtain sufficient diagnostic power. PCa prognostic factors may be used to predict recurrence risk after prostatectomy, transition to an androgen-independent state, or progression-free survival after specific therapies. With very few notable exceptions, prognostic variables have not been correlated to KDM expression. For this reason, we queried two independent gene expression databases to compare KDM expression in different prostate specimens (normal vs. PCa; primary vs. metastatic PCa; low- vs. high-grade; androgen-dependent vs. -independent PCa). In particular, we used a two-step strategy to validate our findings. First, we queried an integrative genomic profiling database of 218 PCa specimens generated at Memorial Sloan-Kettering Cancer Centre (MSKCC) [43]. To validate those findings, KDM genes whose expression was significantly modulated, according to MSKCC database, were also investigated through analysis using the Oncomine database [44]. In order to reduce our false discovery rate, we selected p < 0.01 as a threshold. Table 3 and Figure 2 summarize our results. At first glance, it is evident that most KDM genes emerging from this query have been largely overlooked by previous studies, and may be identified as novel biomarkers. If we decide to prioritize genes significantly associated with clinical variables in both databases, KDM1B, 4C and 5D are the best candidates as potential biomarkers. The only gene with a known function in PCa is KDM4C. As already described, KDM1A and KDM4C co-operate to enhance AR transcriptional activity [32]. KDM4C was also shown to contribute to androgen-independence [31]. Our results confirm the oncogenic role of KDM4C: the MSKCC database indicates that its expression increases with tumor stage, while Oncomine data identify a significant KDM4C overexpression in PCa vs. normal prostate gland (Figure 2) and in grade 3 vs. grade 2 PCa (p = 0.002, fold change 13.125, data not shown). Based on our analysis KDM1B is a putative oncogene, since it is a negative prognostic marker (MSKCC database), and it is specifically overexpressed in CRPC (Oncomine data). Several data from our queries indicate that KDM5D acts as an oncosuppressor in PCa. It’s down-regulation may be involved in tumor-initiation (Figure 2), as well as in increased tumor grade and stage (Table 3). According to those results, its expression predicts longer progression-free survival after prostatectomy (Table 3). Another gene previously associated with cancer is KDM5A. It emerges as a putative prognostic factor from the MSKCC data, although no significant correlation was found in the Oncomine database. Some data from the MSKCC database concordantly suggests an oncogenic function for KDM2B and an oncosuppressive role for KDM4D, although we found no significant correlations using the Oncomine database. In summary, our results indicate that some KDMs may play an important role as novel biomarkers, particularly for prediction of tumor-initiation, progression-free survival and androgen-independent state.

**Table 3:** List of KDM genes significantly up- or down-regulated in PCa clinical subtypes (MSKCC and Oncomine databases)

**Gene**	**Clinical Variable**	**Gene expression**	**P value**	**Putative function**	**Oncomine**
KDM1B	Overall Survival (OS)	Higher predicts shorter OS	0.0045	Oncogene	In agreement
KDM2B	PSA level at diagnosis	Higher predicts higher PSA	0.0070	Oncogene	
	Gleason Score	Higher predicts higher grade	0.0001		
	Lymph Node invasion	Higher predicts invasion	0.0002		
	Metastasis	Higher predicts higher metastatic risk	3.09E-07		
	Recurrence-Free Survival (RFS)	Higher predicts shorter RFS	0.0066		
KDM3B	PSA level at diagnosis	Lower predicts higher PSA	0.0004	Oncosuppressor	In disagreement
	Gleason Score	Lower predicts higher grade	0.0066		
KDM4C	Metastasis	Lower in non-metastatic tumors	0.0086	Oncogene	In agreement
KDM4D	Gleason Score	Higher predicts higher grade	0.0002	Oncogene	
	Lymph Node invasion	Higher predicts invasion	0.0019		
	Recurrence-Free Survival (RFS)	Higher predicts shorter RFS	0.0002		
	Metastasis	Lower in non-metastatic tumors	0.001		
KDM5A	Probability of recurrence after prostatectomy	Higher predicts higher probability	0.002	Oncogene	
KDM5D	Gleason Score	Higher predicts lower grade	0.0035	Oncosuppressor	In agreement
	Lymph Node invasion	Lower predict invasion	0.0036		
	Metastasis	Lower predicts higher metastatic risk	3.7E-5		
	Probability of recurrence after prostatectomy	Lower predicts higher probability	0.0003		
KDM7	Gleason Score	Higher predicts higher grade	0.0016	?	
	Lymph Node invasion	Lower predicts invasion	0.0009		
	Recurrence-Free Survival (RFS)	Higher predicts longer RFS	0.008		

From the MSKCC database, we report all associations with p < 0.01, for the following clinical variables: PSA (prostate specific antigen) levels at diagnosis; Gleason score, based on prostatectomy; lymph node invasion; metastatic risk; recurrence free survival (or probability of recurrence) after prostatectomy; overall survival. Oncomine data are described as “in agreement” if they confirm the oncogene/oncosuppressor function deduced from the MSKCC database. Oncomine thresholds: p < 0.01; fold-change >2.0. Oncomine data are shown in Figure 2. For gene aliases, see Table 1.

**Figure 3:**
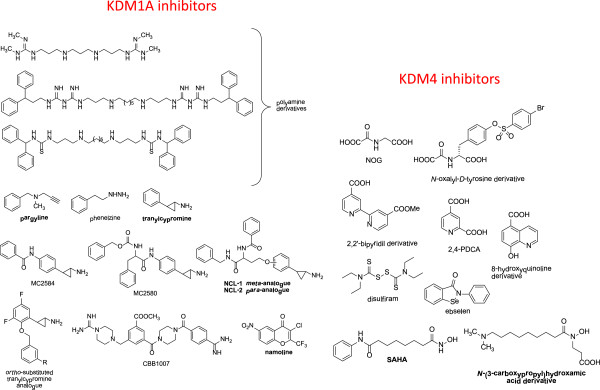
**Chemical structures of KDM inhibitors.** The compounds cited in the text are indicated in bold.

Interestingly, KDMs may be targeted by selective small molecule inhibitors (Figure 3), which are being actively investigated in biochemical and pre-clinical models. KDM inhibitors have been extensively reviewed previously [17]. Here, we will analyze their possible development as anti-PCa agents. Based on its expression pattern and oncogenic function, KDM1A is the most attractive target for PCa therapy. KDM1A inhibitors include substrate analogues, polyamine analogues, and inhibitors of the mono-amino-oxidase (MAO) domain. Since the KDM1A catalytic domain shares homology with neural MAOs [16], pharmacological inhibitors developed as anti-depressive agents have been employed to target cancer cells. Unlike other KDM targeting drugs, MAO inhibitors have already been tested for their pharmacokinetic and toxicity profiles, thus they can be readily translated into the clinics. However, most molecules of this class failed to meet expectations. For example, pargyline was first described as a valuable KDM1A inhibitor in PCa cells after AR induction [25], but further studies failed to confirm this observation [45,46]. Subsequently, tranylcypromine and its analogues proved to more effectively inhibit H3K4 demethylation. In particular, NCL-1 and NCL-2 showed 50% growth inhibiting concentrations ranging from 6 to 17 micromolar when tested on androgen-independent PCa cells [47]. Interestingly, tranylcypromine displayed a safe toxicity profile, even when administered at high-intensity regimens [48]. According to our results (Table 3) and to previously published data [17], tranylcypromine analogues may be particularly effective to prevent PCa recurrence and transition to an androgen-independent state. Future studies should specifically address those points, going beyond the simple measurement of growth inhibitory effects. Clorgyline is another MAO inhibitor which seems to exert anti-proliferative and pro-differentiation activity on high grade-PCa cells [49]. This effect is in part mediated by inhibition of growth factor signalling and down-regulation of EZH2 methyltransferase. The authors did not investigate its effect on KDM1A. Very recently, the γ–pyrone namoline has been described as a reversible KDM1A inhibitor able to inhibit LNCaP cell proliferation, expression of the AR target genes *FKBP5* and *TMPRSS2*, and tumor growth in a mouse PCa model [50].

An interesting approach is the combination of epigenetic therapies. KDM1A and HDAC inhibitors showed synergistic antitumor activity on glioblastoma cells [51]. Since HDAC inhibitors are being tested on PCa patients, this strategy may be explored in this neoplasm too. For example, the HDAC inhibitor vorinostat is a promising epigenetic drug, since it also inhibits EZH2 and H3K4 demethylases at micromolar concentrations [52,53]. The above mentioned combinatorial approach may be tested using KDM1 and JmjC-domain demethylase inhibitors. Unfortunately, a restricted number of potent and selective inhibitors mainly for KDM4 have been developed so far (Figure 3) [54], and none of them have been tested in PCa. The only exception were some hydroxamic acids described as KDM4A/4C inhibitors, that were ineffective on PCa cells as single agents, but displayed synergistic activity in combination with the tranylcypromine analogue NCL-2 [55]. Since different KDMs may act as oncogenes or tumor suppressors, the specificity issue is of crucial importance.

## Tumor-initiating cell epigenetics: is there a role for histone demethylases?

It has been traditionally accepted that all cancer cells are biologically equivalent with every single cell able to form a new tumor. However, during the past few years, mounting evidence has shown that a tumor is composed of heterogeneous cancer cells and only a subset can initiate tumor growth (reviewed in [56]). This specific population, termed tumor initiating cells (TICs), can self-renew and differentiate into non-tumorigenic progeny which make up the tumor bulk. In addition, TICs highly express stem cell specific genes and have been observed to share certain characteristics with normal stem cells [57,58]. This conceptual advance has very important clinical implications, because these cells are considered to be resistant to conventional therapies, such as chemotherapy and irradiation [40,59]. TICs survive after treatment, leading to relapse and acting as the “seeds” for metastasis. Prostate TICs show higher metastatic potential than differentiated cancer cells [60], and some TIC-specific pathways are essential for PCa metastatization [61,62]. TICs are regulated by both genetic and epigenetic factors, such as KDMs. As described, KDM1 triggers gene silencing by removing the methyl groups from H3K4me2 [63,64]. In a very recent study, Wang et al. developed specific bioactive small inhibitors of KDM1A and uncovered that these compounds, or specific KDM1A shRNA, selectively targeted cancer cells with stem cell properties that highly express the stem cell markers OCT4 and SOX2 [65]. However, these inhibitors displayed much lower effects on the growth of non-TICs or normal somatic cells. In correlation with this finding, KDM1A protein levels are highly up-regulated in TICs and in OCT4-positive human PCa tissues, suggesting a critical role for KDM1A and histone H3K4 in TICs. Moreover, high levels of KDM1A are correlated with high-risk tumors and patient relapse after prostatectomy [30]. It has been proposed that a combination of traditional chemo-radiotherapy, plus TIC-specific agents could be optimal for cancer treatment [66]. The first strategy, being effective on the bulk tumor mass, is able to produce an objective response, while the latter should reduce tumor recurrence and progression. KDM1A targeting drugs may therefore emerge as TIC-specific agents in PCa.

H3K9me2 and H3K9me3 are both silencing-associated histone marks. As indicated in Table 2 KDM4C and KDM3A that demethylate these marks are highly expressed in PCa tissues, but their functions remain obscure. In a study on embryonic stem (ES) cells, pluripotency specific transcription factor OCT4 positively regulates KDM4C and KDM3A expression by directly binding to their introns [67]. Interestingly, the depletion of either KDM results in ES cell differentiation, indicating their requirement for the maintenance of self-renewal. In addition, KDM4C activates the expression of Nanog, another important stem cell-specific transcription factor, while KDM3A acts as a positive regulator for pluripotency-associated genes, such as Tcl1, Tcfcp2l1, and Zfp57. Although further investigation is needed to understand how these KDMs contribute to the biology of prostate TICs, we highly suspect that they may play an indispensible role in regulating the stem cell-like properties of prostate TICs. Indeed, epigenetic gene regulation is crucial for maintaining TIC plasticity [9].

During the past few years, more KDMs have been shown to be involved in regulating the biology of the TIC population. As mentioned above, PHF8 [34] and KDM5B [36] are both highly expressed in metastatic prostate tissues and they may play important roles in controlling the invasion and metastasis of PCa cells. Since PCa TICs are more invasive than differentiated cancer cells [68], it is conceivable that these KDMs are involved in TIC metastatic potential, as shown for other histone modifiers [40]. In addition, there is evidence showing that KDMs functionally interact with some other epigenetic machinery, such as the histone methyltransferase EZH2, a Polycomb protein essential for the tumorigenic and metastatic potential of TICs [40,69-71]. The KDM-TIC correlation is still an interesting hypothesis, which needs to be further confirmed. We believe that future studies should investigate KDMs as regulators of prostate TICs and therapeutic targets in pre-clinical models.

## Conclusions

In summary the current data suggest that various KDMs show differential expression in PCa and are likely to play important roles in tumor initiation and progression. Clearly further evaluation of the specific roles of the various KDMs in the bulk tumor, as well as in TICs, is required. Despite our limited knowledge of their functions to date, early studies suggest that targeting KDM activity may provide a new strategy for preventing PCa relapse and progression.

## Competing interests

All authors declare no conflict of interest on the topics covered by this paper.

## Authors’ contribution

FC contributed to study conception, manuscript writing and data analysis. LS contributed to manuscript writing and data analysis. AM contributed to manuscript writing and critically revised the paper. YTC contributed to data analysis. WLF contributed to study conception and critically revised the paper. RD critically revised the paper. CDH contributed to study conception, manuscript writing, data analysis, and critically revised the paper. All authors read and approved the final manuscript.
